# Editorial: Structure and Function of Chloroplasts - Volume II

**DOI:** 10.3389/fpls.2020.620152

**Published:** 2020-11-25

**Authors:** Yan Lu, Lu-Ning Liu, Rebecca L. Roston, Jürgen Soll, Hongbo Gao

**Affiliations:** ^1^Department of Biological Sciences, Western Michigan University, Kalamazoo, MI, United States; ^2^Institute of Systems, Molecular and Integrative Biology, University of Liverpool, Liverpool, United Kingdom; ^3^Department of Biochemistry, University of Nebraska-Lincoln, Lincoln, NE, United States; ^4^Ludwig Maximilian University of Munich, Munich, Germany; ^5^College of Biological Sciences and Biotechnology, Beijing Forestry University, Beijing, China

**Keywords:** chloroplast, envelope, thylakoid, protein import, photosynthesis

As the site of photosynthesis, the chloroplast is responsible for producing all the biomass in plants. It is also a metabolic center for production or modification of many important compounds, such as carbohydrates, purines, pyrimidines, amino acids, fatty acids, precursors of several plant hormones, and many secondary metabolites. The chloroplast also extensively communicates with other parts and organelles of the cell. We were fortunate enough to have submissions from ~100 talented chloroplast researchers. This topic contains 17 papers of which 11 are original research, 4 are reviews or mini-reviews, and one is a perspective.

As the chloroplast is semi-autonomous, the biogenesis, development, division, and partitioning of chloroplasts rely on nuclear-encoded proteins as well. A nuclear-encoded chloroplast-localized translation elongation factor EF-Tu was found to be essential to chloroplast development in the flowering plant *Arabidopsis thaliana* (Liu S. et al.). This prokaryotic-type translation elongation factor also acts cooperatively with the chloroplast translation initiation factor IF3 to control leaf vascular development.

Although most of the chloroplast proteins are encoded by the nuclear genome, the chloroplast genome still contains >100 genes. Efficient transcription of these chloroplast-encoded genes and subsequent translation of protein-coding transcripts is essential to chloroplast function. Nuclear encoded pentatricopeptide repeat proteins (PPRs) have been repeatedly found to be involved in transcription, transcript stabilization, intron splicing, editing, and translation in the chloroplast. Wang et al. discovered that Pigment-Defective Mutant4 (PDM4), a chloroplast P-type PPR protein, plays a crucial role in the expression of chloroplast genes and the development of chloroplasts in Arabidopsis. PDM4 was also found to participate in the splicing of group II introns and possibly the assembly of the large subunit of chloroplast ribosomes. Another chloroplast P-type PPR protein covered by this special issue is Biogenesis Factor required for ATP synthase 2 (BFA2). This protein is capable of binding to the *atpF-atpA* intergenic region in a sequence-specific manner, thus preventing the degradation of the dicistronic *atpH/F* transcript by exoribonucleases (Zhang et al.). The characterizations of two PPR proteins in this special issue demonstrate the importance of PPR proteins in regulating transcription and RNA metabolism in the chloroplast.

Most chloroplast proteins are imported into the chloroplast and delivered to chloroplast subcompartments via complex machinery. Worn-out and damaged chloroplasts and chloroplast components are turned over efficiently to safeguard chloroplast function. Yang et al. reviewed the molecular mechanisms and regulatory pathways of chloroplast protein import and degradation. Relatively little is known about the targeting machinery of tail-anchored proteins, which have stromal-exposed N-terminal domains and a C-terminal transmembrane domain. By studying membrane-specific targeting of two Arabidopsis chloroplast secretory translocase proteins SECE1 and SECE2, which localize to thylakoid membranes and the inner envelope respectively, Anderson et al. discovered that the transmembrane domain and the C-terminal tail of tail-anchored proteins are important for their membrane-specific targeting. Chloroplast stromal chaperone proteins (e.g., HSP90C, HSP70s, and HSP40s) and the GrpE-type nucleotide exchange factors have been proposed to participate in chloroplast protein import, thylakoid integration, assembly and disassembly. Arabidopsis has two nuclear-encoded chloroplast-targeted GrpE proteins: CGE1 and CGE1. Su et al. reported that CGE1 is the main functional homolog among the two and that CGE2 may have a subsidiary or regulatory function.

A chloroplast division site regulator protein, PARC6, was discovered to be critical for chloroplast morphology in pavement and guard cells, and leucoplast morphology in *Arabidopsis* trichome cells (Ishikawa et al.). Analysis of PARC6 fused to a flurorescent protein through confocal microscopysuggested that it forms a ring at the chloroplast division site that changes configuration during chloroplast division. Due to the highly uniform size and shape of leaf epidermal guard cells and the relatively stable number and morphology of chloroplasts in them, leaf epidermal guard cells were recently proposed to be an excellent model system to investigate chloroplast multiplication and partitioning in plants (Fujiwara et al.).

The thylakoid membranes of cyanobacteria and chloroplasts house a series of photosynthetic electron transport complexes. The biogenesis, stabilization, and maintenance of thylakoid membranes require the participation of the inner membrane-associated protein of 30 kDa (IM30), which is also known as the vesicle-inducing protein in plastids 1 (Vipp1). Siebenaller et al. reviewed that the ability of IM30 to form homo-oligomeric protein complexes is crucial to its roles in thylakoid membrane protection and remodeling. In the cyanobacterium *Synechocystis* sp. PCC 6803, thylakoid membranes are reduced in size, function under nitrogen starvation, and are quickly recovered after nitrogen replenishment. Kobayashi et al. reported that the contents of phosphatidylglycerol, an essential phospholipid of photosystem complexes, and glycoglycerolipids, the main constituents of thylakoid membrane lipid bilayers, could be differentially regulated during the recovery from nitrogen starvation. The levels of phosphatidylglycerol recovers quickly after nitrogen is replenished whereas the content of glycoglycerolipids recovers gradually.

Excess light exposure causes oxidative damage to photosynthetic electron transport complexes, especially to the reaction center of photosystem II (PSII). Liu J. et al. reviewed recent advances on reaction center-based approaches for repairing photodamaged PSII and antenna-based approaches for rapid control of PSII light harvesting. Photosynthetic organisms employ these diverse strategies to ensure PSII function under static or fluctuating high light environments. Because of the differences in mobility and environments, land plants are subject to broader high light stress than algae and cyanobacteria. Therefore, land plants developed additional thiol/disulfide-modulating proteins, such as Low Quantum Yield of PSII 1 (LQY1), to repair photodamaged PSII. Wessendorf and Lu found that introducing an Arabidopsis homolog of this protein into the cyanobacterium *Synechocystis* significantly increases the photochemical efficiency of PSII under elevated light conditions. This finding further demonstrated the role of thiol/disulfide-modulating proteins in PSII repair. Photoacclimation of light-dependent reactions involves reversible phosphorylation of thylakoid proteins and redistribution of light-harvesting antenna complexes between PSII and photosystem I (PSI). As a phosphorylation target of state transition kinases 7 and 8, the calcium sensor receptor protein (CAS) was found to play a role in phosphorylation-mediated photoacclimation in Arabidopsis (Cutolo et al.). It was postulated that CAS may modulate photosynthetic function by being phosphorylated in a calcium-dependent manner and/or by influencing the dynamics of chloroplast calcium concentration.

In addition to being a target of calcium signaling, the chloroplast is also an active player in intracellular calcium signaling. Navazio et al. discussed the role of chloroplast calcium signaling under biotic and abiotic stresses and reviewed latest advances in the discovery and characterization of calcium sensors and calcium channels/transporters, especially those that localize to the chloroplast. In the chloroplast, PSI and PSII are major generators of reactive oxygen species (ROS). Although excessive ROS causes oxidative damage, low levels of ROS production triggers retrograde signaling between chloroplasts and the nucleus (Kim), which is beneficial. Beta-carotene and the Executer 1 protein associated with PSII have been proposed to mediate retrograde signaling in the grana core and grana margins, respectively. In addition, the accumulation of ROS-damaged PSII core proteins may trigger a damaged protein response, which induces the expression of protein quality control- and ROS detoxification-related nuclear genes. Furthermore, PSI-driven ROS may inactivate 3′-phosphoadenosine 5′-phosphate (PAP) phosphatase, which leads to PAP accumulation-mediated retrograde signaling. Retrograde signaling also plays vital roles in regulating the expression of nuclear-encoded chloroplast proteins involved in carbohydrate metabolism. For example, the expression of glucose-6-phosphate/phosphate translocator 2 (GPT2) increases rapidly if the growth light is elevated or if starch metabolism is disrupted. The increase of the *GPT2* transcript is preceded by the transcript increases of transcription factors involved in retrograde signaling, including Redox Responsive Transcription Factor 1 (RRTF1) (Weise et al.). Further analyses demonstrated that transcription of the *GPT2* gene requires and the export of triose phosphates from the chloroplast and the expression of RRTF1. In addition to producing sugars and starch, the chloroplast is also a manufacturer for other metabolic important compounds, including purines. The first committing step of de novo purine synthesis is catalyzed by glutamine phosphoribosylpyrophosphate amidotransferases (GPRATs). Cao et al. solved the crystal structure of GPRAT2 and identified the structural differences between *Arabidopsis* GPRAT2 and its bacterial homologs. GPRAT2 was previously identified as the target of a small molecule, DAS34. The inhibition mechanism of DAS34 was characterized at the structural level in this study. This work sheds light on further development of herbicides targeting GPRATs.

Altogether, this volume of the Research Topic provides exiting works in the area of the structure, functions and maintenance of chloroplasts, ranging from the biogenesis and development of chloroplasts to gene transcription, protein synthesis and turnover, metabolism, as well as the dynamics, signaling and regulation of chloroplast functions including photosynthesis. With the efforts of many researchers worldwide, the frontiers of this topic keep evolving at a rapid pace.

## Author Contributions

All authors listed have made a substantial, direct and intellectual contribution to the work, and approved it for publication.

## Conflict of Interest

The authors declare that the research was conducted in the absence of any commercial or financial relationships that could be construed as a potential conflict of interest.

